# Substantial improvements not seen in health behaviors following corner store conversions in two Latino food swamps

**DOI:** 10.1186/s12889-016-3074-1

**Published:** 2016-05-11

**Authors:** Alexander N. Ortega, Stephanie L. Albert, Alec M. Chan-Golston, Brent A. Langellier, Deborah C. Glik, Thomas R. Belin, Rosa Elena Garcia, Ron Brookmeyer, Mienah Z. Sharif, Michael L. Prelip

**Affiliations:** Department of Health Management and Policy, Dornsife School of Public Health, Drexel University, 3215 Market Street, Nesbitt Hall, Room 335, Philadelphia, PA 19104 USA; Department of Community Health Sciences, Fielding School of Public Health, University of California, Los Angeles, Los Angeles, CA 90095 USA; Department of Biostatistics, Fielding School of Public Health, University of California, Los Angeles, Los Angeles, CA 90095 USA

**Keywords:** Corner store, Food deserts, Food environment, Food supply, Healthy food availability, Obesity, Urban health, Latinos, Hispanic Americans, Food policy

## Abstract

**Background:**

The effectiveness of food retail interventions is largely undetermined, yet substantial investments have been made to improve access to healthy foods in food deserts and swamps via grocery and corner store interventions. This study evaluated the effects of corner store conversions in East Los Angeles and Boyle Heights, California on perceived accessibility of healthy foods, perceptions of corner stores, store patronage, food purchasing, and eating behaviors.

**Methods:**

Household data (*n* = 1686) were collected at baseline and 12- to 24-months post-intervention among residents surrounding eight stores, three of which implemented a multi-faceted intervention and five of which were comparisons. Bivariate analyses and logistic and linear regressions were employed to assess differences in time, treatment, and the interaction between time and treatment to determine the effectiveness of this intervention.

**Results:**

Improvements were found in perceived healthy food accessibility and perceptions of corner stores. No changes were found, however, in store patronage, purchasing, or consumption of fruits and vegetables.

**Conclusions:**

Results suggest limited effectiveness of food retail interventions on improving health behaviors. Future research should focus on other strategies to reduce community-level obesity.

**Electronic supplementary material:**

The online version of this article (doi:10.1186/s12889-016-3074-1) contains supplementary material, which is available to authorized users.

## Background

Disparities have been documented in the prevalence of overweight and obesity among low-income and ethnic minority populations. For example, in the United States, 78 % of Latinos are overweight or obese compared with 67 % of non-Latino whites [1]. Several public health intervention strategies have been implemented and tested to reduce disparities in obesity. These include individual-level programs to improve physical activity and dietary habits [2–4], as well as community-level social marketing and education campaigns [5, 6].

Recently, there has been an increase in interventions to improve eating behaviors through supportive changes to the built environment. These initiatives have largely been concentrated in low-income neighborhoods that have a disproportionate prevalence of chronic diseases, as well as poor access to affordable healthy food. Strategies to improve access to healthy foods have included introducing farmers’ markets to communities, changing restaurant menu offerings, and improving access to grocery and corner stores that sell affordable fresh fruits and vegetables [7–9].

Findings on the impact of corner store interventions to improve the food environment in low-income communities have been mixed [10, 11]. For example, an intervention to improve healthy food access and marketing in corner stores and markets in Baltimore found positive changes in healthy food purchasing and preparation among patrons and individuals from local community organizations [12]. A review of small-store intervention studies found that interventions were able to increase the availability of healthy items and improve knowledge about health and nutrition [10]. Findings were mixed, however, in terms of sales and purchasing of healthy food items, perceived availability of healthy items, and behavioral intentions to purchase healthy items [10]. Similarly, a small store intervention in North Carolina aimed at promoting sales of fruits and vegetables had mixed success. Results indicated that stores increased the availability of vegetables but not fruit, and there were no differences in consumption of either vegetables or fruits as a result of the intervention [8]. In another intervention, the nutritional content of purchases remained unchanged following a large-scale effort to increase the availability of healthier products in Philadelphia corner stores [13].

Despite these mixed findings, food environment interventions have gained considerable traction among federal and state policymakers, as well as private funders. For example, in 2011, the Federal Government announced the Healthy Food Financing Initiative, a $400 million program to improve access to healthy foods and to eliminate food deserts in the U.S. [14]. Similarly, the California FreshWorks fund is a $272 million public-private partnership loan fund that finances new and upgraded grocery stores to improve healthy food access to low-income communities [15]. The high cost of food environment interventions underscores the need for rigorous studies to evaluate best practices for reducing community-level obesity.

### Overview of corner store conversions

The components of corner store interventions are varied, but they typically include changing the interior and exterior of stores, modifying floor plans, installing new shelving and refrigeration units, and increasing availability of affordable fresh fruits and vegetables. Some interventions, however, have not included core elements of formative research, such as assessing community health practices and needs, determining residents’ perceptions of food environments, and understanding neighborhood contexts. This allows for program developers to implement interventions that are accepted by community residents, while also facilitating the development of community-tailored social marketing campaigns, both of which can help sustain the demand for the newly available products. Moreover, few interventions have explicitly included store owner training on important practices such as managing inventories and negotiating with wholesalers. In addition to programmatic limitations, evaluations have been hampered by small sample sizes, lack of comparison stores, short evaluation time frames, and examination only of changes among store patrons without attention to potential changes at the community level [10, 11].

### The intervention: *Proyecto MercadoFRESCO*

The intervention, *Proyecto MercadoFRESCO*, has been described in greater detail elsewhere [16]. In brief, the intervention was based in two urban communities in Los Angeles (LA) County, California, East LA and Boyle Heights. These neighboring communities are comprised of majority Mexican-American populations. In particular, 97.1 % of the 2010 Census population in East LA was Latino, making it the proportionally largest Latino population in the US [17]. Both of these communities have a high prevalence of overweight and obesity. For example, nearly 77 % of East LA residents are overweight or obese [18]. Both communities have been characterized as food swamps, within which residents are faced with a disproportionate number of places to purchase unhealthy foods (e.g., fast food restaurants, taco stands, and corner stores) and have fewer options for healthy food purchasing than in more affluent areas.

The project was community-engaged in order to create a sense of “ownership” within the community. A broad range of community partners including business owners, schools, community-based organizations, local politicians, and a community health center participated on the community advisory board (CAB) and helped guide the project on all aspects of the intervention design, implementation and evaluation. Additionally, the study included a formative research phase in which ten focus groups, with a total of 92 community members, were held to elicit residents’ perceptions about corner stores and their views about the food environment and facilitators and barriers to purchasing, preparing, and consuming healthy foods. In general, formative research demonstrated that community residents had negative perceptions regarding the quality, healthfulness, and affordability of foods sold in local corner stores. Thus, from the outset, the intervention sought to improve community perceptions regarding corner stores in order to increase patronage, purchasing, and consumption of healthy foods sold at the stores.

Four stores were recruited to be intervention stores, and four additional stores were selected as comparisons. One of the intervention stores stopped implementation activities during the early stages of the planned conversion effort at the request of the store owner. At the analysis stage, this store was treated as part of the comparison group, resulting in three intervention stores and five comparison stores. The comparison stores were located at least a mile away from intervention stores and were separated by a major freeway, in order to limit the potential for contamination effects.

Intervention stores received a comprehensive “makeover” to both the interior and exterior of the locations including new signage and paint, security upgrades, store layout alterations, product placement, and social marketing promotions for healthful eating. Each store was also provided refrigeration equipment at no cost to display a variety of newly available fresh fruits and vegetables. The intervention specifically focused on increasing the availability of fruits and vegetables; however, storeowners were autonomous in decisions regarding the variety and quantity of produce in an effort to match these items to the preferences and needs of local residents. The increase was substantial, with most stores adding at least a dozen new fruits and vegetables to their inventory.

In addition to the store renovation, owners received training on business practices provided by a former corner store owner with experience and expertise in converting stores into healthier outlets. They received training on issues relevant to the procurement and handling of affordable fresh fruits and vegetables including minimizing waste, meeting community demand, removing spoiled inventory, and developing business relationships with produce wholesalers and local farmers' markets. This training was included to increase store owner engagement, maintain profitability, and enhance sustainability. There was also a multi-component social marketing campaign implemented in the study neighborhoods. After stores were converted, they were monitored for fidelity to ensure that stores stocked fresh fruits and vegetables and that those items were of good quality. Comparison stores were also monitored throughout the study period to ensure that no comparable improvements were made to the appearance of the store or their merchandise.

This paper will present findings from this community-engaged, multi-level corner store intervention project. Baseline and follow-up findings from a survey of community residents are examined with regard to perceptions of the food environment and corner stores as well as patronage, food purchasing, and consumption behaviors.

## Methods

A household survey of neighborhood residents was administered to assess the impact of the intervention at the community-level. In-depth information regarding the study protocol can be found elsewhere [16]. The study was approved by the Institutional Review Board at UCLA, and all study participants provided informed consent.

The study employed a repeated cross-sectional evaluation design. In-person household interviews were conducted in the neighborhoods surrounding the converted and comparison stores to test the main research question, “can community-level changes be achieved by improving the food environment through corner store conversions.” Based on *a priori* power calculations, the target sample size was 1000 residential addresses at baseline (i.e., 125 around each store) and an additional 1000 at follow-up. Sampling was conducted using a multi-stage process. First, catchment areas were constructed for each of the intervention and comparison stores. Catchment areas included the blocks closest in proximity to each of the stores. Blocks were selected until there were roughly 300 households in a catchment area, which would allow for enough participants based on expected occupancy, screening, and response rates. Next, households were randomly sampled from each catchment area and approached for participation. Finally, the adult identified as the primary food purchaser and preparer for the household was invited to participate. Respondents from homes surrounding converted stores comprise the intervention group, while respondents from homes around comparison stores make up the comparison group.

At baseline, 1035 community residents completed a household survey, for an American Association for Public Opinion Research (AAPOR) response rate (i.e. number of completed interviews divided by the number of individuals who were eligible) of 80 %. Twelve to 24-months following baseline interviews, the same residential addresses were again approached for participation. In order to be eligible at follow-up, household residents had to live in the neighborhood for at least one year. This minimum residency requirement necessitated additional households to be sampled in order to achieve roughly equal number of interviews. During the second round of data collection, 1052 individuals completed the survey for an AAPOR household follow-up response rate of 75 %. Sixty-three percent of baseline respondents were re-interviewed at follow-up.

The interviews were administered face-to-face using computer-assisted personal interviewing (CAPI) and took approximately one and a half hours to complete. Respondents had the choice of completing the interview in Spanish or English and received a $25 gift card to a large local market for participation.

### Measures

Demographic measures included in the study were sex, age (in years), marital status, nativity status (US-born/foreign-born), Mexican heritage (yes/no), primary language spoken at home (English-only, bilingual, or Spanish-only), education (in years), and participation in the Special Supplemental Nutrition Program for Women, Infants, and Children or Supplemental Nutrition Assistance Program (yes/no).

This study focuses on five outcomes of interest: (1) perceived accessibility of healthy foods, (2) perception of corner stores, (3) corner store patronage, (4) food purchasing, and (5) eating behaviors.

#### Accessibility of healthy foods

Four questions measured perceived accessibility of healthy foods: (a) you have a convenient place to buy healthy food, (b) healthy food is too expensive, (c) it is hard to find places in your neighborhood where you can buy healthy foods, and (d) the healthy foods sold in your neighborhood are of low quality. Respondents were asked to indicate if these statements were true or false. Although these questions were newly developed, they were consistent in content with a previous study [19]. These items were explored as separate outcomes estimating individuals’ perceptions of neighborhood healthy food accessibility.

#### Perceptions of corner stores

Fifteen items assessed participants’ perceptions about corner stores. These items were developed by the research team *de novo* and represent major themes identified during the formative research phase of the study, such as convenience, price, quality, variety, and customer service. Individuals were first asked if there is a local corner store where they usually shop. Those individuals responding affirmatively were subsequently asked to name the store, and the 15 items measuring perceptions were then asked about that specific store. Individuals that did not have a usual corner store were asked about their general perceptions of corner stores. Respondents were asked to indicate whether they believed statements about corner stores were true or false. Responses indicating a positive perception towards a corner store were coded as 1 while negative and “don’t know” responses were coded as 0. A score summing the number of positive perceptions across the 15 questions was also calculated (range: 0–15; Cronbach alpha: 0.83).

#### Store patronage

Store patronage was counted as affirmative when a respondent reported having purchased food on at least one occasion at an intervention or comparison store.

#### Food purchasing

In order to assess food purchasing behaviors, participants were asked to estimate the total number of dollars spent per week on all food, as well as how much per week was spent on canned, frozen, or fresh fruits and vegetables. A percentage of the total amount spent on fruits and vegetables was then calculated by dividing the amount spent on produce by the total spent on all food.

#### Eating behaviors

Participants were also asked to estimate how many servings of fruits and how many servings of vegetables they eat each day. These two questions were taken from the validated Food Behavior Checklist [20] and were summed to reflect total daily fruit and vegetable intake.

### Statistical analyses

The analyses conducted for this study included participants with data at either or both baseline and follow-up. Additionally, only those individuals with data for all variables of interest were included in the final analyses, yielding an analytic sample of 1686 household interviews (baseline *n* = 795; follow-up *n* = 891). Comparability in demographic characteristics between the full sample and the subsample of participants with no missing data was assessed. There were no statistically significant differences between the groups, suggesting that the subsample is a reasonably good representation of the sample as a whole.

All analyses were performed using STATA 14.0. Summary statistics were performed for all variables. Chi-squared tests were used to assess whether the intervention and comparison groups improved from baseline to follow-up for the following binary outcome variables: healthy food accessibility, corner store characteristics, and store patronage. Independent sample t-tests were used to identify statistically significant differences in the summary perception score, food purchasing, and food consumption between time points. Differences between baseline and follow-up were calculated for both the comparison group and the intervention group for all variables of interest. To test for statistically significant differences in these change scores, the interaction of intervention status and time in regression models was used.

Multiple logistic regression models were fitted to estimate odds ratios for perceptions of food accessibility for time, intervention status, and the interaction between time and intervention status while controlling for sex, age (years), nativity status, language spoken at home, education (years), and food assistance. Multiple linear regressions modeling the summary corner store perception score, food purchasing, and food consumption were performed controlling for the same variables as previously described.

Finally, sensitivity analyses were conducted to contrast the findings using the sample of all available respondents with the findings from the subset of individuals who completed interviews at both baseline and follow-up (*n* = 650). Using conditional logistic regressions for binary variables and linear regressions with random effects for continuous variables, these analyses accounted for the within-person associations that arise from repeated measures on an individual.

## Results

At baseline for both the intervention and comparison samples, the majority of participants were female, younger than 50, married or living with partner, foreign-born, of Mexican-heritage, had less than a high school education, and did not receive food assistance (see Table 1). The only difference between the groups was that a smaller proportion of respondents in the intervention sample spoke only Spanish as compared to the comparison sample.

**Table 1:** Characteristics of Proyecto MercadoFRESCO Sample (*N* = 1686)

	Baseline Intervention Percent or Mean (SD)	Baseline Comparison Percent or Mean (SD)	Follow-up Intervention Percent or Mean (SD)	Follow-up Comparison Percent or Mean (SD)
	*N* = 313	*N* = 482	*N* = 323	*N* = 568
Characteristics				
Sex				
Male	21.7	22.4	17.3	21.0
Female	78.3	77.6	82.7	79.0
Age (Years)	44.4 (15.5)	44.3 (15.9)	47.1 (14.8)	46.4 (15.4)
Marital Status^a^				
Single	23.6	21.8	22.4	21.9
Married/With Partner	56.2	58.5	58.1	60.9
Separated/Divorced/Widowed	20.1	19.7	19.6	17.3
Nativity				
U.S. Born	37.4	35.9	34.7	33.5
Foreign Born	62.6	64.1	65.3	66.5
Mexican Heritage^b^				
Yes	89.2	84.9	95.3	88.5**
No	10.8	15.1	4.7	11.5
Language Spoken at Home				
English Only	13.7	14.9**	12.7	8.6**
English and Spanish	56.2	45.2	67.5	62.1
Spanish Only	32.0	39.8	19.8	29.2
Language of Interview				
English	43.1	39.6	43.0	40.3
Spanish	56.9	60.4	57.0	59.7
Education (years)	9.9 (4.1)	10.3 (3.9)	10.1 (4.3)	10.0 (4.1)
Food Assistance^c^				
Yes	26.8	31.5	28.2	31.5
No	73.2	68.5	71.8	68.5

NOTE: Some percentages may not sum to 100 due to rounding. Significant differences between intervention and comparison in categorical variables were tested at baseline and follow-up using chi-squared tests. Significant differences between intervention and comparison in continuous variables were tested at baseline and follow-up independent sample t-tests tests

**p <0 .01

^a^Data were only available for 332 (99.8 %) for the follow-up intervention group and 567 (99.8 %) observations for the follow-up control group due to missing data

^b^Data were only available for 306 (97.8 %) for the baseline intervention group, 469 (97.3 %) observations for the baseline control group, 318 (98.4 %) for the follow-up intervention group, and 555 (97.7 %) for the follow-up control group due to missing data

^c^Participation in Special Supplemental Nutrition Program for Women, Infants or Children (WIC) or Supplemental Nutrition Assistance Program (SNAP)

At follow-up participants had similar demographic characteristics. The only differences between the samples at that time were in the intervention sample a larger percentage were of Mexican heritage and a smaller percentage were Spanish-only speakers than the comparison sample.

### Perceptions of healthy food accessibility

As Table 2 shows, there was a non-statistically significant increase between baseline and follow-up in the percentage of intervention neighborhood residents who reported having a convenient place to buy healthy food, while there was a statistically significant improvement among residents of comparison neighborhoods. Participants from both communities were less likely to perceive challenges in finding places within their neighborhoods to buy healthy foods at follow-up. No changes were found between baseline and follow-up regarding perceived expense or quality of healthy food in either neighborhood, even though the majority of respondents indicated that healthy food was too expensive and that the quality of food sold was poor. Additionally, no intervention effects were found when testing for an interaction between time and intervention status (see Additional file 1: Table S1).

**Table 2:** Perceptions about Food Accessibility by Intervention Status and Time

	Intervention Percent		Comparison Percent		Percent Difference (Follow-up – Baseline)	
	Baseline	Follow-up	Baseline	Follow-up	Intervention	Comparison
	(*N* = 313)	(*N* = 323)	(*N* = 482)	(*N* = 568)		
You have a convenient place where you can buy healthy food	83.4	88.2	77.8	88.2***	4.8	10.4
Healthy food is too expensive	52.4	53.6	55.2	53.0	1.2	−2.2
It’s hard to find places in your neighborhood where you can buy healthy foods	39.3	31.3*	39.0	33.1*	−8.0	−5.9
The healthy foods sold in your neighborhood are low quality	56.2	51.7	63.5	57.6	−4.5	−5.9

NOTES: Significant differences were tested between intervention baseline and follow-up using chi-squared tests, comparison baseline and follow-up using chi-squared tests, and percent difference (follow-up – baseline) for intervention and comparison using a Wald test on the interaction term of a logistic regression (more details can be seen in Additional file 1: Table S1). This Wald test can be thought of as testing whether the relative change (on an odds ratio scale) is the same in the intervention and comparison groups

*p < 0.05,***p < 0.001

### Perceptions of corner stores

Improvements were found in perceptions of corner stores from baseline to follow-up for both the intervention and comparison samples (see Table 3). A larger proportion of participants at follow-up reported that corner stores sell a wide variety of fresh, frozen, and canned fruits and vegetables, as well as healthy foods in general. Only in the comparison sample did perceptions of fruit quality improve, while only in the intervention sample did perceptions of vegetable quality improve.

**Table 3:** Perceptions about Corner Stores and Patronage by Intervention Status and Time

	Intervention		Comparison		Percent or Mean Difference (Follow-up – Baseline)	
	Percent or Mean (SD)		Percent or Mean (SD)			
	Baseline	Follow-up	Baseline	Follow-up	Intervention	Comparison
	(*N* = 313)	(*N* = 323)	(*N* = 482)	(*N* = 568)		
Corner Store Characteristics						
Corner stores sell a wide variety of fresh fruits	8.9	26.3***	18.5	26.6**	17.4	8.1
Corner stores sell a wide variety of fresh vegetables	10.9	35.6***	19.5	29.6***	24.7	10.1
Corner stores sell a wide variety of frozen or canned fruits	27.2	36.2*	31.1	42.3***	9.0	11.2***
Corner stores sell a wide variety of frozen or canned vegetables	25.9	35.3*	30.5	41.7***	9.4	11.2**
Fresh fruits sold at corner stores are not of poor quality^a^	40.0	45.5	44.2	51.4*	5.5	7.2
The fresh vegetables sold at corner stores are of good quality	22.7	44.0***	25.1	30.3	21.3	5.2
Corner stores sell healthy food	34.2	51.4***	37.6	45.2*	17.2	7.6
Corner stores are not dirty^b^	60.1	77.4***	66.2	68.8	17.3	2.6
Corner stores are not dangerous^c^	70.0	83.9***	75.5	75.2	13.9	−0.3
Corner stores have good customer service	73.5	85.4***	70.5	75.0	11.9	4.5
I can get information about nutrition and healthy eating at corner stores	10.2	28.5***	12.9	16.2	18.3	3.3
Corner stores sell traditional Latino food ingredients	77.0	83.6*	76.6	83.5**	6.6	6.9***
The staff at corner stores speaks my language	86.3	92.0*	82.8	90.7***	5.7	7.9
Food sold at corner stores is not expensive^d^	20.4	22.6	28.2	28.0	2.2	−0.2
It is convenient to shop at corner stores	47.6	50.5	49.4	52.3	2.9	2.9
Overall Beliefs About Corner Stores Score (Range: 0-15	6.1 (3.4)	8.0 (3.6)***	6.7 (3.4)	7.6 (3.6)***	1.9	1.0
Corner Store Patronage						
Shops at 1 or more study stores	41.5	46.7	28.2	23.4	5.2	−4.8***

NOTES: Significant differences in binary variables were tested between intervention baseline and follow-up using chi-squared tests, comparison baseline and follow-up using chi-squared tests, and percent difference (follow-up – baseline) for intervention and comparison using a Wald test on the interaction term of a logistic regression (more details can be seen in Additional file 2: Table S2). This Wald test can be thought of as testing whether the relative change (on an odds ratio scale) is the same in the intervention and comparison groups. Significant differences in continuous variables were tested between intervention baseline and follow-up using independent sample t-tests, comparison baseline and follow-up using independent sample t-tests tests, and mean difference (follow-up – baseline) for intervention and comparison using a F-test on the interaction term of a linear regression (more details can be seen in Additional file 3: Table S3)

*p < 0.05, **p < 0.01, ***p < 0.001

^a^Question was reverse coded. Original statement was “Fresh fruits sold at corner stores are of poor quality”

^b^Question was reverse coded. Original statement was “Corner stores are dirty”

^c^Question was reverse coded. Original statement was “Corner stores are dangerous”

^d^Question was reverse coded. Original statement was “Food sold at corner stores is expensive”

Corner store characteristics associated with store conversions improved over time for the intervention group only. Specifically, perceptions regarding the cleanliness of corner stores, safety of corner stores, quality of customer service, and ability to get information about healthy eating and nutrition all improved between baseline and follow-up. Participants from both samples reported increased availability of culturally appropriate Latino ingredients and language concordance between customers and staff at follow-up.

The intervention effects of the individual items measuring corner store perceptions were mixed, with three significant interactions showing more improvements in comparison store, but they mainly showed patterns of non-significance (See Additional file 2: Table S2). The summary index score, which is a more robust measure than the individual items, showed marked improvements over time in both samples (See Additional file 3: Table S3). No significant intervention effect was found when testing for an interaction between time and intervention status.

### Store patronage

The proportion who reported patronizing one of the study stores remained consistent between baseline and follow-up (see Table 3) for both intervention and comparison samples. A larger percentage of respondents from the intervention neighborhoods reported shopping at these study stores at both times.

### Food purchasing

No differences were observed between baseline and follow-up for the intervention or comparison samples in the percent of total dollars spent on fruits and vegetables per week (see Table 4). Moreover, the interaction between intervention condition and time was not statistically significant, suggesting that the intervention had no effect on food purchasing (see Additional file 3: Table S3).

**Table 4:** Food Purchasing and Fruit and Vegetable Consumption by Time and Intervention Status

	Intervention		Comparison		Mean Difference (Follow-up – Baseline)	
	Mean (SD)		Mean (SD)			
	Baseline	Follow-up	Baseline	Follow-up	Intervention	Comparison
	(*N* = 313)	(*N* = 323)	(*N* = 482)	(*N* = 568)		
Dollars spent on food per week^a^	131.6 (64.0)	140.6 (126.0)	123.1 (66.4)	134.8 (74.8)**	9.0	11.7
Dollars spent on fruits and vegetables per week^a^	46.2 (28.4)	49.8 (33.9)	44.6 (29.7)	47.2 (32.6)	3.6	2.6
Percent of dollars spent on fruits and vegetables	36.5 (16.7)	38.1 (17.6)	37.7 (18.0)	36.9 (18.7)	1.6	−0.8
Servings of fruits and vegetables consumed each day	4.4 (2.0)	4.2 (2.1)	4.5 (2.4)	4.8 (2.3)	−0.2	0.3

NOTES: Significant differences in continuous variables were tested between intervention baseline and follow-up using independent sample t-tests, comparison baseline and follow-up using independent sample t-tests tests, and mean difference (follow-up – baseline) for intervention and comparison using a F-test on the interaction term of a linear regression (more details can be seen in Additional file 3: Table S3)

**p < 0.01

^a^The significance of the mean difference was not tested, as this variable was only of interest to calculate percent of dollars spent on fruits and vegetables

### Fruit and vegetable consumption

Table 4 shows that consumption of fruits and vegetables did not change over time among respondents from either sample. A test for an intervention effect was also non-significant (see Additional file 3: Table S3).

### Sensitivity analyses

In separate sensitivity analyses that are not shown, results indicated that the main findings presented above were robust regardless if the data were limited to those participants who had responses at both time points, accounting for correlation between repeated measurements from the same respondent. The fact that different analytical strategies yielded qualitatively similar results provide reassurance that the main findings of the study were not artifacts of analytical choices.

## Discussion

Corner store conversions, particularly in urban and rural food deserts and swamps, have emerged as a popular strategy to improve healthy food access, despite limited evidence on their effectiveness [10, 11]. This study, in two neighboring Latino food swamps, found that regardless of intervention status, perceptions of food accessibility and corner stores improved over time, but effects on patronage, purchasing and consumption of healthy foods were non-significant. In general, the mixed findings are consistent with the results of other studies [8, 10, 11, 13].

While there were modest improvements in perceptions of the food environment, these changes did not appear to be attributable to the intervention alone. One explanation is that other local initiatives were underway in the two communities during the study period. Initiatives have included providing health education and promoting access to healthy food [21, 22]. The combination of initiatives might have collectively improved perceptions of the food environment over time. Nevertheless, because improvements were not found in key behavioral outcomes associated with obesity, these findings challenge the notion that simply changing the food environment will improve eating behaviors. Alternatively, urban food swamps may be so densely-populated with low-cost, unhealthy food options that modest interventions at a limited number of retailers may be insufficient to improve health behaviors and outcomes.

This study makes a number of contributions to the science of food environment interventions. Most notably, the study used community engagement to understand perceptions, needs and demands about food accessibility and healthy eating, which informed the design, implementation, and evaluation of the intervention. Moreover, the intervention had a variety of community partners who participated in the conversions, marketing campaigns, and evaluation [16].

In addition to the community-wide social marketing, the study benefited from the ability to provide store owners with technical assistance to facilitate purchasing and selling of fresh produce. In short, because the intervention supplemented the physical transformations of the stores with these additional resources and support systems, the conversions approximate the ‘best case scenario’ of what might be expected of interventions in a limited number of small food stores’ offerings.

Another advantage was that this study utilized comparison stores, which helped to answer the larger policy question as to whether this type of intervention will lead to community-level changes. The study also allowed sufficient time to detect changes, with up to two years between baseline and follow-up data collection. Finally, the survey had a relatively large sample size compared with most prior studies. This study was among the most rigorously designed and implemented interventions aimed at modifying the built environment, yet no significant changes in dietary behavior were found.

Our findings are consistent with similar types of interventions that have also found that altering the food environment does not necessarily lead to improved healthy eating behaviors. For example, a grocery store intervention in North Philadelphia raised awareness about healthy eating but did not impact fruit and vegetable intake [19]. Similarly, a Whole Foods grocery store that was introduced into a Detroit food desert did not improve patronage or consumption of healthy food items among local residents; rather, the store was largely supported by professionals who worked in the area [23]. A recent study detailing the results of a new supermarket in the South Bronx, New York, found no observable changes in fruit and vegetable consumption or overall diet quality one year after the store opening [24]. These and other findings suggest that simply improving access to healthy foods does not necessarily translate into improved dietary behaviors within the community.

Similarly, a 2008 LA zoning regulation banned new or remodeled fast food restaurants in South LA, another urban food swamp; this ban did not result in lowering the prevalence of obesity as expected [25]. The policy directive, however, lacked the inclusion of important public health intervention initiatives such as raising awareness about healthy eating, reducing the number of venues that sell unhealthy foods, and offering economic disincentives, such as taxes on unhealthy food, to promote community health.

Reducing disparities in obesity has proven to be difficult. The public health field continues to grapple with the best strategies for using the ecological framework in health disparities interventions, including implementing multi-level intervention projects that have large and sustainable population impact. Albeit limited, evidence has demonstrated that these approaches can in fact be effective. For example, a community-based participatory research intervention in Somerville, Massachusetts resulted in significant reductions in child and parent obesity [26, 27]. This intervention had comprehensive partnerships that allowed for effective planning, implementation, and dissemination. Food environment interventions need to be comprehensive in scope and address multiple components of the social determinants of health framework [28] through public-private partnerships, with local, regional, and national policy support, in order to improve healthy eating and ultimately decrease the prevalence of obesity among the most vulnerable populations.

### Limitations

The study had a range of limitations. First, this study relied on self-reports of behaviors that are subject to recall and reporting biases. Second, due to factors not under the control of the investigative team, one of the intervention stores dropped out during the early stages of its conversion, leading to it being considered as a comparison store in the analyses. Third, we did not have sales data, as it was much more difficult than anticipated to collect cash-register data from these small independently owned stores. Fourth, because the study prioritized interviewing the primary food purchaser and preparer, the community sample is majority female, which may limit the generalizability of the findings beyond primary household food purchasers and preparers.

## Conclusions

The potential of corner store conversions to improve eating behaviors in urban food swamps does not by itself seem promising. Although this type of food environment intervention is important, it needs to be part of broader public health initiatives that include the combination of individual-, community-, and policy-level solutions. As the obesity epidemic is complex, it will take efforts on multiple fronts as well as sufficient time to reduce obesity and its related health consequences.

### Ethics approval and consent to participate

All study protocols were approved by the UCLA Institutional Review Board. Written informed consent was obtained from all study participants.

### Availability of data and materials

Participant level data are available from the last author MLP.

## Additional files

Additional file 1: Table S1. Logistic Regression Predicting Food Accessibility (*N* = 1686). (DOC 34 kb) Additional file 2: Table S2. Logistic Regression Predicting Perceptions about Corner Stores and Patronage (*N* = 1686). (DOC 47 kb) Additional file 3: Table S3. Regression Models Predicting Purchasing, Consumption and Corner Store Perceptions (*N* = 1686). (DOC 38 kb)

## Abbreviations

AAPOR: American Association for Public Opinion Research
CAPI: computer-assisted personal interviewing.
LA: Los Angeles
