# Supplementary material for: Substantial improvements not seen in health behaviors following corner store conversions in two Latino food swamps
Source: BMC Public Health. 2016 May 11;16:389. doi: 10.1186/s12889-016-3074-1 (PMC4864998; doi:10.1186/s12889-016-3074-1)
Supplement: Additional file 1: Table S1. — Logistic Regression Predicting Food Accessibility (N = 1686). (DOC 34 kb) [file 12889_2016_3074_MOESM1_ESM.doc]

| **Supplemental Table 1: Logistic Regression Predicting Food Accessibility (N=1,686)** | | | | |
| --- | --- | --- | --- | --- |
|  | **You have a convenient place where you can buy healthy food** | **Healthy food is too expensive** | **It’s hard to find places in your neighborhood where you can buy healthy foods** | **The healthy foods sold in your neighborhood are low quality** |
| **Unadjusted Model** | **OR (95% CI)** | **OR (95% CI)** | **OR (95% CI)** | **OR (95% CI)** |
| Time | 2.2 (1.5, 3.2)*** | 1.0 (0.8, 1.3) | 0.7 (0.6, 1.0)* | 0.8 (0.6, 1.0) |
| Intervention | 1.4 (1.0, 1.8) | 1.0 (0.8, 1.2) | 1.0 (0.8, 1.2) | 0.8 (0.6, 1.0)* |
| Time*Intervention | 0.7 (0.5, 1.2) | 1.0 (0.7, 1.3) | 1.0 (0.7, 1.4) | 1.0 (0.7, 1.4) |
| Intercept | 3.6 (2.9, 4.4)*** | 1.2 (1.0, 1.4)* | 0.7 (0.5, 0.8)*** | 1.7 (1.4, 2.0)*** |
| **Adjusted**  **Model^1^** | **AOR (95% CI)** | **AOR (95% CI)** | **AOR (95% CI)** | **AOR (95% CI)** |
| Time | 2.1 (1.5, 3.1)*** | 1.0 (0.7, 1.2) | 0.7 (0.6, 1.0)* | 0.8 (0.6, 1.1) |
| Intervention | 1.4 (1.0, 1.9)* | 1.0 (0.8, 1.2) | 0.9 (0.7, 1.2) | 0.7 (0.6, 0.9)* |
| Time*Intervention | 0.7 (0.5, 1.2) | 1.0 (0.7, 1.3) | 1.0 (0.7, 1.4) | 1.0 (0.7, 1.4) |
| Intercept | 2.1 (0.8, 5.3) | 1.1 (0.6, 2.3) | 0.9 (0.4, 2.0) | 4.0 (1.9, 8.3)*** |
| NOTES: The reference categories were baseline and comparison community for time and intervention respectively.  ^1^ Adjusted for gender, age (years), nativity status, language use, education (years), and food assistance | | | | |
