# Supplementary material for: Substantial improvements not seen in health behaviors following corner store conversions in two Latino food swamps
Source: BMC Public Health. 2016 May 11;16:389. doi: 10.1186/s12889-016-3074-1 (PMC4864998; doi:10.1186/s12889-016-3074-1)
Supplement: Additional file 2: Table S2. — Logistic Regression Predicting Perceptions about Corner Stores and Patronage (N = 1686). (DOC 47 kb) [file 12889_2016_3074_MOESM2_ESM.doc]

| **Supplemental Table 2: Logistic Regression Predicting Perceptions about Corner Stores and Patronage (N=1,686)** | | | | |
| --- | --- | --- | --- | --- |
|  | **Corner stores sell a wide variety of fresh fruits** | **Corner stores sell a wide variety of fresh vegetables** | **Corner stores sell a wide variety of frozen or canned fruits** | **Corner stores sell a wide variety of frozen or canned vegetables** |
| **Unadjusted Model** | **OR (95% CI)** | **OR 95% CI)** | **OR (95% CI)** | **OR (95% CI)** |
| Time | 2.3 (1.7,3.2)*** | 2.4 (1.8,3.3)*** | 2.2 (1.7,2.9)*** | 2.1 (1.6,2.8)*** |
| Intervention | 0.8 (0.6,1.1) | 0.9 (0.7,1.3) | 1.1 (0.8,1.4) | 1.0 (0.8,1.3) |
| Time*Intervention | 0.8 (0.5,1.1) | 1.0 (0.7,1.4) | 0.5 (0.4,0.7)*** | 0.6 (0.4,0.8)** |
| Intercept | 0.2 (0.1,0.2)*** | 0.2 (0.2,0.2)*** | 0.4 (0.3,0.5)*** | 0.4 (0.3,0.5)*** |
|  | **Fresh fruits sold at corner stores are not of poor quality^1^** | **The fresh vegetables sold at corner stores are of good quality** | **Corner stores sell healthy food** | **Corner stores are not dirty^2^** |
| **Unadjusted Model** | **OR (95% CI)** | **OR 95% CI)** | **OR (95% CI)** | **OR (95% CI)** |
| Time | 1.5 (1.1,1.9)** | 1.7 (1.3,2.2)*** | 1.6 (1.2,2.1)*** | 1.3 (1.0,1.7)* |
| Intervention | 0.9 (0.7,1.1) | 1.3 (1.0,1.7)* | 1.1 (0.8,1.4) | 1.0 (0.8,1.3) |
| Time*Intervention | 0.8 (0.6,1.1) | 1.1 (0.8,1.5) | 1.0 (0.7,1.4) | 1.2 (0.9,1.7) |
| Intercept | 0.8 (0.7,0.9)** | 0.3 (0.2,0.3)*** | 0.6 (0.5,0.7)*** | 1.8 (1.5,2.1)*** |
|  | **Corner stores are not dangerous^3^** | **Corner stores have good customer service** | **I can get information about nutrition and healthy eating at corner stores** | **Corner stores sell traditional Latino food ingredients** |
| **Unadjusted Model** | **OR (95% CI)** | **OR 95% CI)** | **OR (95% CI)** | **OR (95% CI)** |
| Time | 1.1 (0.9,1.5) | 1.6 (1.2,2.1)** | 1.7 (1.2,2.5)** | 2.0 (1.4,2.8)*** |
| Intervention | 1.0 (0.8,1.3) | 1.5 (1.2,2.0)** | 1.3 (0.9,1.8) | 1.2 (0.9,1.6) |
| Time*Intervention | 1.3 (0.9,1.9) | 0.9 (0.6,1.3) | 1.2 (0.8,1.9) | 0.6 (0.4,0.9)* |
| Intercept | 2.8 (2.3,3.3)*** | 2.2 (1.8,2.6)*** | 0.1 (0.1,0.2)*** | 3.1 (2.5,3.7)*** |
|  | **The staff at corner stores speaks my language** | **Food sold at corner stores is not expensive^4^** | **It is convenient to shop at corner stores** | **Shops at 1 or more study stores** |
| **Unadjusted Model** | **OR (95% CI)** | **OR 95% CI)** | **OR (95% CI)** | **OR (95% CI)** |
| Time | 1.9 (1.3,2.8)** | 0.9 (0.7,1.2) | 1.3 (1.0,1.6) | 1.4 (1.0,1.8)* |
| Intervention | 1.2 (0.9,1.7) | 0.6 (0.5,0.8)** | 1.0 (0.8,1.3) | 3.0 (2.3,3.9)*** |
| Time*Intervention | 1.0 (0.6,1.8) | 1.4 (1.0,1.9) | 0.8 (0.6,1.1) | 0.5 (0.4,0.7)*** |
| Intercept | 4.9 (3.9,6.2)*** | 0.4 (0.3,0.5)*** | 0.9 (0.8,1.1) | 0.3 (0.3,0.4)*** |
| NOTES: The reference categories were baseline and comparison community for time and intervention respectively.  * p<.05, ** p<.01, *** p<.001  ^1^ Question was reverse coded. Original statement was “Fresh fruits sold at corner stores are of poor quality”  ^2^ Question was reverse coded. Original statement was “Corner stores are dirty”  ^3^ Question was reverse coded. Original statement was “Corner stores are dangerous”  ^4^ Question was reverse coded. Original statement was “Food sold at corner stores is expensive” | | | | |

\
