# Supplementary material for: Substantial improvements not seen in health behaviors following corner store conversions in two Latino food swamps
Source: BMC Public Health. 2016 May 11;16:389. doi: 10.1186/s12889-016-3074-1 (PMC4864998; doi:10.1186/s12889-016-3074-1)
Supplement: Additional file 3: Table S3. — Regression Models Predicting Purchasing, Consumption and Corner Store Perceptions (N = 1686). (DOC 38 kb) [file 12889_2016_3074_MOESM3_ESM.doc]

| **Supplemental Table 3: Regression Models Predicting Purchasing, Consumption and Corner Store Perceptions (N=1,686)** | | | | | | | | | |
| --- | --- | --- | --- | --- | --- | --- | --- | --- | --- |
|  | **Overall Perceptions About Corner Stores Score (Range: 0-15)** | | | **Percent of total dollars spent on fruits and vegetables x100** | | | **Number of servings of fruits and vegetables consumed each day** | | |
| **Unadjusted Model** | b | SE | p | b | SE | p | b | SE | p |
| Time | 1.4 | 0.2 | < 0.001 | 0.2 | 1.2 | 0.83 | 0.2 | 0.1 | 0.13 |
| Intervention | 0.1 | 0.2 | 0.65 | 0.1 | 1.1 | 0.92 | -0.2 | 0.1 | 0.11 |
| Time*Intervention | -0.3 | 0.3 | 0.21 | -0.2 | 1.4 | 0.88 | -0.3 | 0.2 | 0.08 |
| Intercept | 6.4 | 0.1 | < 0.001 | 37.2 | 0.8 | < 0.001 | 4.6 | 0.1 | < 0.001 |
| **Adjusted Model^1^** | b | SE | p | b | SE | p | b | SE | p |
| Time | 1.4 | 0.2 | < 0.001 | 0.6 | 1.2 | 0.63 | 0.2 | 0.1 | 0.12 |
| Intervention | 0.1 | 0.2 | 0.73 | 0.4 | 1.1 | 0.71 | -0.2 | 0.1 | 0.13 |
| Time*Intervention | -0.3 | 0.3 | 0.33 | -0.5 | 1.4 | 0.70 | -0.3 | 0.2 | 0.05 |
| Intercept | 8.1 | 0.6 | < 0.001 | 31.7 | 3.2 | < 0.001 | 3.9 | 0.4 | < 0.001 |
| NOTES: The reference categories were baseline and comparison community for time and intervention respectively.  * p<.05, ** p<.01, *** p<.001  ^1^ Adjusted for gender, age (years), nativity status, language use, education (years), and food assistance | | | | | | | | | |
